# The impact of intuitive eating *v*. pinned eating on behavioural markers: a preliminary investigation

**DOI:** 10.1017/jns.2020.25

**Published:** 2020-08-12

**Authors:** Jane Ogden, Elina Pavlova, Hollie Fouracre, Frances Lammyman

**Affiliations:** 1 School of Psychology, University of Surrey, Guildford GU2 7XH, UK; 2 School of Nutrition, University of Surrey, Guildford GU2 7XH, UK

**Keywords:** Intuitive eating, Pinned eating, Eating, Trial, Behaviour

## Abstract

Two promising strategies to manage eating behaviour are intuitive eating (IE; following hunger) and pinned eating (PE; ignoring hunger/eating at specific times of the day). This study compared IE and PE on behavioural markers. Participants (*n* 56) were randomly assigned to IE (*n* 28) or PE (*n* 28) and given instructions to follow for 1 week. Drive to eat, behaviour, behavioural intentions and self-efficacy were measured at baseline and follow-up. Participants also evaluated their specific intervention. Comparable changes over time were found for both conditions for many measures. Significant conditions by time interactions were found for healthy snacking, total self-efficacy and self-efficacy for weight loss: those following IE showed an increase in each of these outcomes compared to those following PE who showed no change. The IE group found their intervention more useful than those following PE. Further research is needed to build on these preliminary findings.

## Introduction

Poor eating behaviour can lead to a number of health problems including obesity, diabetes, heart disease, malnutrition and reduced life expectancy. Yet evidence indicates that interventions to improve eating behaviour have only limited effectiveness. Over the past few years, two new potential interventions have emerged. These are intuitive eating (IE) and pinned eating (PE), which offer two contrasting ways to improve eating behaviour which will now be described.

The concept of IE was introduced in 1995 and emphasises the need to follow internal cues and eat only when physically hungry^(1,2)^. Preliminary evidence suggests that IE may improve body image and self-efficacy, is associated with lower BMI and cholesterol, and may promote long-term weight maintenance^(2–4)^. Given our current obesogenic environment and the wide availability of food, however, much research indicates that the ability to identify biological signals of hunger may be blunted and that food intake is governed by other factors such as environmental triggers, emotions and cognitions. For example, some studies have explored the notion of eating in the absence of hunger which has been found to be less apparent in children, more common in young women and linked with elements of disordered eating^(5,6)^. Likewise, research indicates that food intake is influenced by the words used to describe food, where and how food is eaten, how food is presented and that people consume more when distracted by external influences such as the TV, music or social interaction^(7–9)^. The notion of IE is therefore promising as a strategy to improve eating behaviour, but it may be hard to implement given the world we live in.

In contrast to a focus on internal cues, an alternative strategy to weight management involves an emphasis on external cues, specifically time, known as PE. Research shows that an increasing number of people ‘Eat on the go’ and that there is a shift towards snacking rather than consuming meals^(10–12)^. Furthermore, this shift away from designated meals is associated with a reduction in vegetable intake, overeating and subsequent weight gain^(5,6)^. In contrast, research indicates that much food intake is already governed by external cues^(8,9)^ and that limiting snacking, introducing meal planning and meal timing can reduce body weight and food intake^(13–15)^, Furthermore, time-restricted feeding which limits the window when feeding can occur has also been shown to be beneficial to weight^(15)^. Likewise, endocrinological studies indicate that eating at set times each day leads to hormonal entrainment whereby ghrelin is released only around the pre-programmed times, thus controlling physical hunger^(16)^. PE therefore involves a reliance on external cues to eat and encourages eating at specific times of the day. This negates the requirement to identify internal cues of hunger and promotes meal consumption rather than snacking. It is possible, however, that PE may contribute to eating problems in line with research on dietary restraint^(17)^.

To conclude, interventions to improve eating behaviour have limited effectiveness. Two new promising approaches are IE and PE yet to date there is limited data on their impact. The aim of this study was to carry out a preliminary evaluation the relative impact of IE compared to PE on behavioural markers focusing on the drive to eat, behaviour and self-efficacy and to assess participants’ own evaluations of trying to adhere to these contrasting approaches. Due to the absence of previous research, no specific hypotheses were made concerning how these two interventions may impact on the outcome variables.

## Methods

### Design

A randomised controlled trial was employed in which participants were randomly assigned to either IE or PE conditions. This study was conducted according to the guidelines laid down in the Declaration of Helsinki, and all procedures involving human subjects/patients were approved by the University of Surrey Ethics Committee (1372-PSY-18RS). Written informed consent was obtained from all subjects/patients.

### Sample

Participants were recruited via advertisements across a University campus and completed an online screening questionnaire. Exclusion criteria were pregnant/breastfeeding; history of eating disorders; specific dietary requirements and taking medication. Inclusion criteria were aged over 18 years. The advertisement asked ‘Do you want help managing your food intake?’ Eligible participants (*n* 56) completed baseline measures and then were randomly assigned to either the IE (*n* 28) or PE (*n* 28) conditions and given instructions to follow for 1 week and then completed. Participants were contacted 1 week later to complete the follow-up measures and the evaluation.

### The interventions

Participants were given instructions as follows based on the existing research on IE^(1–3)^ and PE^(13,14)^.

### Intuitive eating

#### We would like you to try to eat when PHYSICALLY HUNGRY

People can feel physical and emotional hunger. Physical hunger is a physical feeling of emptiness accompanied by signals such as stomach rumbling, weakness, irritability and low energy. Emotional hunger can be triggered by sadness, loneliness, boredom and stress.

For the next week, we would like you to follow your physical hunger only. See the points below to help you eat only when you are physically hungry:

Whenever you feel hungry ask yourself: Am I feeling empty? Is my stomach rumbling? Do I feel weak, irritable and low in energy? If YES, then eat; Rate the question ‘How physically hungry am I?’ ranging from 1 (starving) to 5 (actually quite full). Ideally you should eat between 1 and 3; Have a glass of water and pause, if you still feel hungry have something to eat; If you decide that you are not physically hungry distract yourself by listening to music or calling a friend or going for a walk!

Evidence shows that eating only when physically hungry can reduce overeating

### Pinned eating

#### We would like you to try PINNED EATING

Like a clock on the wall, your body also has a clock of its own. Having set meal times can make us better at controlling our hunger which can lead to a better relationship with food. By only eating at set times each day, we teach our body to expect food at these times which allows us to ignore hunger for other reasons. For the following week, we would like you only to eat at set times of the day to pin your eating to these specific times. See the points below to help you develop the habit of eating at specific times. Chose three times per day when you are going to eat in the range of breakfast 06.00–09.00, lunch 12.00–14.00 and dinner 18.00–20.00. Write these times down and stick to them; DO NOT snack in-between these three mealtimes; Make sure you stick to the three meal times you have chosen

Evidence shows that pinning meals to specific times can reduce overeating.

They were asked to print out these instructions and place them somewhere visible for the next week.

### Measures

Participants completed measures at baseline and after 1 week. Reliability was assessed using Cronbach's *α* where appropriate.

### Profile characteristics

At baseline, all participants recorded their sex, age, ethnicity, occupation, weight, height and their perceived weight status. They also completed the restrained eating scale^(18)^.

### Outcome measures

The following outcome measures were assessed at baseline and 1 week follow-up.

**Drive to eat:** This was assessed for the past week as follows: (i) **hunger** (3 items, e.g. ‘I could always eat’ *t*1 *α* 0⋅6; *t*2 *α* 0⋅7); (ii) **fullness** (3 items, e.g. ‘My stomach is full’ *t*1 *α* 0⋅6; *t*2 *α* 0⋅7); (iii) **desire to eat** (3 items, e.g. ‘I am looking forward to eat’ *t*1 *α* 0⋅8; *t*2 *α* 0⋅8); (iv) **physical hunger** (3 items, e.g. ‘My stomach had felt empty’ *t*1 *α* 0⋅7; *t*2 *α* 0⋅7); (v) **emotional eating** (3 items, e.g. ‘I have felt guilty after eating’ *t*1 *α* 0⋅9; *t*2 *α* 0⋅9); (vi) **overeating** (3 items, e.g. ‘I have eaten too much’ *t*1 *α* 0⋅9; *t*2 *α* 0⋅9). All items were measured on a Likert scale ranging from ‘not at all’ (1) to ‘very much’ (5). These items were based on those used in previous research to assess changes in the drive to eat in a number of different situations^(8,10)^.

**Behaviour:** Behaviour was assessed for the past week in terms of: (i) **Snacking behaviour**: (healthy (10 items, e.g. ‘Fruit’ *t*1 *α* 0⋅7; *t*2 *α* 0⋅7); unhealthy (10 items, e.g. ‘Chips’ *t*1 *α* 0⋅7; *t*2 *α* 0⋅7) assessed using a Likert scale ranging from ‘Never’ (1) to ‘More than 3 times per day, every day’ (5); (ii) **Adherence to** IE and PE was assessed for the past week: [IE (3 items, e.g. ‘Assessed my level of physical hunger’ *t*1 *α* 0⋅6; *t*2 *α* 0⋅6); PE (3 items, e.g. ‘Not snacked throughout the day’ *t*1 *α* 0⋅8; *t*2 *α* 0⋅9)]. All items were assessed on a 5-point Likert scale ranging from ‘Never’ (1) to ‘Always’ (5). The measure of snacking was based on a scale used previously in a number of different research settings^(19)^. The adherence measure was designed specifically for this intervention and reflected the literature describing IE and PE^(e.g. 1–3,13,14)^.

**Behaviour intentions and self-efficacy:** (i) **Behavioural intentions (BI)** was assessed for aspects of PE and IE [IE (3 items, e.g. ‘Assess my level of physical hunger before eating’ *t*1 *α* 0⋅9; *t*2 *α* 0⋅9); PE (3 items, e.g. ‘Set my 3 meal times and stick to it’ *t*1 *α* 0⋅8; *t*2 *α* 0⋅8)]. All items were assessed on a 5-point Likert scale ranging from ‘Never’ (1) to ‘Always’ (5). (ii) **Self-efficacy (SE)** was assessed in terms of: (1) weight (3 items, e.g. ‘Lose weight’ *t*1 *α* 0⋅8; *t*2 *α* 0⋅9); (2) eat less (3 items, e.g. ‘Prevent overeating’ *t*1 *α* 0⋅6; *t*2 *α* 0⋅6); (3) adherence (2 items, e.g. ‘Follow these recommendations’ *t*1 *α* 0⋅8; *t*2 *α* 0⋅8). Both behavioural intentions and self-efficacy scales were assessed for Right Now using a 5-point Likert scale ranging from ‘Not at all’ (1) to ‘very much’ (5). A total self-efficacy scale was also computed (*t*1 *α* 0⋅9; *t*2 *α* 0⋅9). These measures were developed specifically for this study to reflect behavioural intentions and self-efficacy specific to the interventions being tested.

### Evaluation

At the follow-up, participants completed a measure of their evaluation of the usefulness of their intervention. This consisted of six items (The instructions were clear; The changes were easy to follow; The changes fitted into my lifestyle; I managed to follow the instructions; I felt that the changes were useful; I intend to carry on trying to eat this way in the future) which were rated for the past week using a 5-point Likert scale ranging from ‘Not at all’ (1) to ‘Very much’ (5). A total score was computed (*α* 0⋅8).

Mean scores were computed for each subscale.

### Data analysis

Sample size was calculated using G Power to detect a small-to-medium effect size in the difference in self-reported behaviour between the two groups based on previous experimental work^(e.g. 7,8,10)^. The data were analysed to describe participant profile characteristics and to assess differences in these by condition using the *t*-test and the chi-square test (*x*^2^); to explore the impact of condition on changes in drive to eat, behaviour, behavioural intentions and self-efficacy using repeated-measures ANOVA and *post hoc* tests; to assess differences by condition in the evaluation using *t*-tests.

## Results and discussion

### Participant profile characteristics

Participant profile characteristics are shown in Table 1.

**Table 1.** Participant profile characteristics

	All participants (*N* 56)	IE (*N* 28)	PE (*N* 28)	*P*/*T*/*X*^2^
Age	M = 27⋅1	M = 26	M = 28⋅2	*t* = 0⋅105
	sd = 7⋅8	sd = 9⋅8	sd = 10⋅4	*P* = 0⋅29
Sex	m = 15 (26⋅8%)	m = 8 (22⋅1%)	m = 6 (21⋅4%)	*X*^2^ = 0⋅82
	f = 41 (73⋅2%)	f = 19 (67⋅9%)	f = 22 (78⋅6)	*P* = 0⋅37
Ethnicity	White = 44 (78⋅6%)			
	Black = 2 (3⋅6%)	W = 21(75%)	W = 23 (82⋅1%)	*X*^2^ = 2⋅09
	Asian = 10 (17⋅9%)	O = 7 (25%)	O = 5 (17⋅9%)	*P* = 0⋅35
	Other 0 (0%)			
Occupation	Student = 33 (58⋅9%)	S = 16 (57⋅1%)	S = 17 (67%)	
	Working = 21 (37⋅5%)	W = 11 (39⋅3%)	W = 10 (35⋅7%)	*X*^2^ = 0⋅078
	Unemployed = 2 (3⋅6%)	U = 1 (3⋅6%)	U = 1 (3⋅6%)	*P* = 0⋅962
BMI (kg/m^2^)	M = 23⋅8	M = 23⋅2	M = 24⋅4	*t* = −0⋅9
	sd = 4⋅3	sd = 3⋅7	sd = 4⋅9	*P* = 0⋅33
Perceived weight	Underweight = 3 (5⋅3%)	U = 1 (3⋅6%)	U = 2 (7⋅1%)	
	Healthy = 40 (71⋅4%)	H = 20 (71⋅4%)	H = 20 (71⋅4%)	*X*^2^ = −1⋅66
	Over = 12 (21⋅4%)	Ow = 7 (25%)	Ow = 5 (17⋅9%)	*P* = 0⋅64
	Obese = 1 (1⋅8%)	Ob = 0 (0%)	Ob = 1 (3⋅6%)	
Restrained eating	M = 2⋅6	M = 2⋅58	M = 2⋅63	*t* = −0⋅189
	sd = 0⋅93	sd = 1⋅81	sd = 1⋅05	*P* = 0⋅851

Bold denotes significant difference (*P* < 0⋅05).

M, mean; m, male; f, female; IE, intuitive eating; PE, pinned eating.

The majority of participants were white, healthy weight females aged about 27 years who described a moderate level of restrained eating. No differences were found between the two conditions, indicating that randomisation was effective.

### The impact of condition

The impact of the condition on the key outcome variables was assessed using repeated-measures ANOVA.

#### Drive to eat

The impact of condition on changes in drive to eat is shown in Table 2.

**Table 2.** Impact of condition of drive to eat (mean (sd))

	IE (*N* 28)		PE (*N* 28)		ME		Time × Condition
	*t*_1_	*t*_2_	*t*_1_	*t*_2_	Time	Condition	
Hunger	2⋅94 (1⋅09)	2⋅59 (1⋅01)	2⋅67 (0⋅72)	2⋅67 (0⋅89)	f = 1⋅9	f = 0⋅19	f = 2⋅2
					*P* = 0⋅17	*P* = 0⋅67	*P* = 0⋅14
					*η*^2^ = 0⋅03	*η*^2^ = 0⋅005	*η*^2^ = 0⋅04
Fullness	2⋅34 (0⋅63)	2⋅34 (0⋅68)	2⋅46 (0⋅66)	2⋅29 (0⋅57)	f = 0⋅83	f = 0⋅06	f = 0⋅83
					*P* = 0⋅37	*P* = 0⋅81	*P* = 0⋅17
					*η*^2^ = 0⋅02	*η*^2^ = 0⋅001	*η*^2^ = 0⋅03
Desire to eat	3⋅52 (0⋅14)	3⋅08 (0⋅88)	3⋅29 (0⋅80)	3⋅08 (0⋅84)	**f = 12⋅99**	f = 0⋅28	f = 1⋅55
					***P* = 0⋅001**	*P* = 0⋅601	*P* = 0⋅22
					***η*^2^ =** **0⋅92**	*η*^2^ = 0⋅005	*η*^2^ = 0⋅03
Emotional eating	2⋅45 (1⋅27)	1⋅9 (1⋅00)	2⋅2 (0⋅92)	1⋅80 (0⋅62)	**f = 11⋅79**	f = 0⋅37	f = 0⋅27
					***P* = 0⋅002**	*P* = 0⋅43	*P* = 0⋅61
					***η*^2^ =** **0⋅17**	*η*^2^ = 0⋅012	*η*^2^ = 0⋅005
Physical hunger	2⋅22 (0⋅83)	2⋅21 (0⋅73)	2⋅2 (0⋅65)	2⋅3 (0⋅73)	f = 0⋅02	f = 0⋅37	f = 2⋅18
					*P* = 0⋅89	*P* = 0⋅54	*P* = 0⋅15
					*η*^2^ = 0⋅0001	*η*^2^ = 0⋅007	*η*^2^ = 0⋅039
Overeating	2⋅63 (1⋅26)	2⋅07 (0⋅90)	2⋅44 (1⋅06)	2⋅04 (0⋅92)	**f = 12⋅24**	f = 0⋅19	f = 0⋅38
					***P* = 0⋅001**	*P* = 0⋅66	*P* = 0⋅54
					***η*^2^ =** **0⋅82**	*η*^2^ = 0⋅004	*η*^2^ = 0⋅007

Bold denotes significant difference (*P* < 0⋅05).

f, female; IE, intuitive eating; PE, pinned eating; ME, main effect.

The results showed a main effect of time for drive to eat, emotional eating and overeating indicating a decrease in these measures over the course of the week regardless of condition. There were no significant main effects of condition nor any condition by time interactions.

#### Behaviour

The impact of condition on snacking behaviour and adherence is shown in Table 3.

**Table 3.** Impact of condition on behaviour (mean (sd))

	IE		PE		ME		
	*t*_1_	*t*_2_	*t*_1_	*t*_2_	Time	Condition	Time × Condition
Healthy snacking	2⋅6 (0⋅64)	2⋅79 (0⋅61)	2⋅36 (0⋅86)	2⋅14 (0⋅73)	f = 0⋅02	**f = 6⋅73**	**f = 5⋅50**
					*P* = 0⋅87	***P* = 0⋅01**	***P* = 0⋅02**
					*η*^2^ = 0⋅001	***η*^2^ =** **0⋅005**	***η*^2^ =** **0⋅093**
Unhealthy snacking	1⋅99 (0⋅68)	1⋅77 (0⋅53)	1⋅87 (0⋅54)	1⋅6 (0⋅48)	**f = 12⋅75**	f = 1⋅09	f = 0⋅14
					***P* = 0⋅001**	*P* = 0⋅30	*P* = 0⋅70
					***η*^2^ =** **0⋅191**	*η*^2^ = 0⋅02	*η*^2^ = 0⋅003
Adherence (PE)	2⋅05 (1⋅11)	2⋅6 (1⋅25)	2⋅26 (1⋅1)	3⋅14 (1⋅25)	**f = 20⋅39**	f = 1⋅89	f = 1⋅1
					***P* = 0⋅0001**	*P* = 0⋅18	*P* = 0⋅30
					***η*^2^ =** **0⋅27**	*η*^2^ = 0⋅03	*η*^2^ = 0⋅02
Adherence (IE)	2⋅38 (0⋅82)	3⋅16 (0⋅79)	2⋅34 (0⋅90)	2⋅72 (0⋅92)	**f = 18⋅79**	f = 2⋅21	f = 1⋅48
					***P* = 0⋅0001**	*P* = 0⋅14	*P* = 0⋅22
					***η*^2^ =** **0⋅25**	*η*^2^ = 0⋅03	*η*^2^ = 0⋅02

Bold denotes significant difference (*P* < 0⋅05).

f, female; IE, intuitive eating; PE, pinned eating; ME, main effect.

The results showed a main effect of time for unhealthy snacking, adherence to PE and adherence to IE, indicating that over the course of the week participants reported a reduction in unhealthy snacking and adherence to their intervention regardless of their condition. The results also showed a main effect of condition for healthy snacking with those in the IE condition snacking significantly more than PE. Further, the results showed a significant time by condition interaction for healthy snacking. *Post hoc* tests showed that whereas the IE group significantly increased their healthy snacking (*t* −2⋅67, *P* = 0⋅013), those in the PE group showed no change (*t* 1⋅38; *P* = 0⋅18).

#### Behavioural intentions and self-efficacy

The impact of condition on behavioural intentions and self-efficacy is shown in Table 4.

**Table 4.** Impact of condition on behavioural intentions and self-efficacy (mean (sd))

	IE		PE		ME		
	*t*_1_	*t*_2_	*t*_1_	*t*_2_	Time	Condition	Time × Condition
Behavioural intentions IE	3⋅76 (0⋅95)	3⋅60 (1⋅01)	3⋅61 (1⋅22)	3⋅53 (1⋅01)	f = 0⋅42	f = 0⋅24	f = 0⋅03
					*P* = 0⋅52	*P* = 0⋅62	*P* = 0⋅84
					*η*^2^ = 0⋅008	*η*^2^ = 0⋅005	*η*^2^ = 0⋅09
Behavioural intentions PE	2⋅92 (1⋅2)	2⋅92 (1⋅1)	3⋅71 (1⋅1)	3⋅09 (1⋅0)	f = 0⋅64	f = 3⋅52	f = 3⋅64
					*P* = 0⋅6	*P* = 0⋅06	*P* = 0⋅06
					*η*^2^ = 0⋅001	*η*^2^ = 0⋅06	*η*^2^ = 0⋅06
Total self-efficacy	2⋅72 (0⋅72)	3⋅3 (0⋅96)	3⋅08 (0⋅89)	2⋅84 (0⋅88)	f = 0⋅76	f = 0⋅22	**f = 4⋅4**
					*P* = 0⋅83	*P* = 0⋅63	***P* = 0⋅04**
					*η*^2^ = 0⋅01	*η*^2^ = 0⋅004	***η*^2^ = 0⋅07**
Self-efficacy ‘eat’	2⋅92 (0⋅87)	3⋅23 (0⋅98)	3⋅17 (0⋅66)	3⋅01 (0⋅82)	f = 0⋅11	f = 0⋅02	f = 1⋅2
					*P* = 0⋅73	*P* = 0⋅88	*P* = 0⋅28
					*η*^2^ = 0⋅002	*η*^2^ = 0⋅001	*η*^2^ = 0⋅002
Self-efficacy ‘weight’	2⋅73 (0⋅82)	3⋅48 (1⋅32)	3⋅26 (1⋅10)	2⋅75 (0⋅90)	f = 2⋅5	f = 0⋅48	**f = 7⋅09**
					*P* = 0⋅61	*P* = 0⋅49	***P* = 0⋅01**
					*η*^2^ = 0⋅005	*η*^2^ = 0⋅009	***η*^2^ = 0⋅116**
Self-efficacy ‘adherence’	2⋅51 (0⋅89)	3⋅12 (1⋅02)	2⋅73 (1⋅03)	2⋅81 (0⋅86)	f = 2⋅63	f = 0⋅10	f = 1⋅46
					*P* = 0⋅11	*P* = 0⋅75	*P* = 0⋅23
					*η*^2^ = 0⋅04	*η*^2^ = 0⋅002	*η*^2^ = 0⋅02

Bold denotes significant difference (*P* < 0⋅05).

f, female; IE, intuitive eating; PE, pinned eating; ME, main effect.

No significant main effects of time or condition were found for measures of behavioural intention or self-efficacy. There were, however, significant condition by time interactions for total self-efficacy and self-efficacy for weight loss. *Post hoc* tests indicated that whereas those in the IE condition showed an increased in their total self-efficacy (*t* −2⋅84; *P* = 0⋅009) and their self-efficacy for weight loss (*t* −2⋅48; *P* = 0⋅02), those in the PE condition showed no change for either of these measures (*t* 0⋅57; *P* = 0⋅580 and *t* 1⋅39; *P* = 0⋅17, respectively). There was also a trend for a significant time by condition interaction for behaviour intentions to carry out PE (*P* = 0⋅06). The means indicate that whereas those in the IE group showed no change, those in the PE indicated a decrease in their intentions.

#### Evaluation

The results were analysed for the individual evaluation items and showed no differences between conditions for statements relating to the instructions being clear, the changes being easy to follow, the changes fitting to their lifestyle and the changes being useful. However, those in the IE condition reported greater scores for being able to follow the instructions (mean 3⋅43; sd 1⋅2) compared to those in the PE condition (mean 2⋅71; sd 1⋅3), (*t* 2⋅11; *P* = 0⋅04). Further those in the IE condition also reported greater intention to carry on eating this way in the future (mean 3⋅71; sd 1⋅2) compared to those in the PE condition (mean 3⋅04; sd 1⋅2), (*t* 2⋅16; *P* = 0⋅04). The results also indicated that overall the IE group found the intervention more useful (mean 3⋅5; sd 0⋅8) than the PE group (mean 3⋅0; sd 0⋅8), (*t* 2⋅9; *P* = 0⋅04).

This preliminary study aimed to compare the relative impact of IE which involved following internal bodily signals of hunger and fullness and PE which was based on external cues and included pinning meals to specific set times each day.

The results showed no differences between IE and PE for changes in measures of drive to eat including hunger and fullness, emotional eating and overeating. The results, however, did show some differences for total self-efficacy and self-efficacy specific to weight loss. In particular, whereas those in the IE condition reported an increase in both these measures of self-efficacy, those following PE showed no change. This provides support for previous research, indicating that IE may have a beneficial impact on aspects of eating control^(2,3)^. Furthermore, those following IE also reported an increase in healthy snacking, whereas those in the PE group showed no change. This may further illustrate the benefits of IE over PE if healthy snacking has health benefits and has replaced unhealthy snacking^(3)^. In contrast, however, this result may indicate that IE encourages snacking as opposed to the consumption of meals which in turn could contribute to weight gain. This could illustrate relative benefits of PE over IE as a means to limit snacking encourage meal consumption and illustrate how focusing on external rather than internal cues may a useful approach to eating control^(15,20)^. Finally, the results also provide some insights into participants’ responses to the two different forms of intervention and show that IE was considered more useful overall, that the IE instructions were easier to follow and that the participants showed greater intention to carry on with the IE approach in the future. This was also reflected in a trend for a reduction in participants’ intentions to adhere to PE over time, suggesting that this form of intervention was less feasible than IE.

This is a preliminary study aiming to contrast two novel approaches to eating behaviour. As such there are some limitations with this study that need to be considered. The main limitation is that the intervention and follow-up were short term. Nonetheless, short-term studies provide some relevant data as immediate influences of the intervention can be detected and they provide valuable insights before undertaking a more comprehensive analysis. In addition, the outcome measures were based on self-report and only proxy measures of health which are open to bias and social desirability. Finally, it is likely that IE and PE are experienced differently by different populations. Further research is therefore needed to explore the longer term implications of these two interventions and whether they are sustainable over time. In addition, future research could explore the impact of IE and PE on more objective outcomes such as food intake, body weight and aspects of health status. Likewise, a larger study is required to enable analysis of sub-groups by factors such as body weight and health status and an assessment of who benefits most from which approach.

To conclude, improving eating behaviour could have wide ranging benefits for health. To date, however, interventions show only limited effectiveness. The present study aimed to evaluate two novel approaches and contrasted a focus on internal cues (IE) with a focus on external cues (PE). These preliminary results indicate that, in the short term, IE may be easier to adopt and could improve self-efficacy. It may, however, also increase healthy snacking. Further research is needed to test the impact of these two approaches in the longer term and with more objective outcomes such as food intake and body weight.
